# From Binge Scrolling to Problematic Technology Use: Fear of Missing Out as a Self-Regulatory Mediator

**DOI:** 10.3390/bs16050802

**Published:** 2026-05-18

**Authors:** Alex James Holte, Ava B. Wise, Andrew J. Nixon

**Affiliations:** 1 Psychology Department, University of Wisconsin-La Crosse, La Crosse, WI 54601, USA; 2 Behavioral Science Department, University of Cincinnati—Blue Ash College, Blue Ash, OH 45236, USA

**Keywords:** binge-scrolling, fear of missing out, problematic smartphone use, problematic social media use

## Abstract

Binge-scrolling, the consecutive viewing of digital content, may contribute to maladaptive technology use behaviors such as Problematic Smartphone Use (PSU) and Problematic Social Media Use (PSMU). Grounded in Compensatory-Internet Use Theory (CUIT), the present study examined whether Fear of Missing Out (FoMO) mediated the relationships between binge-scrolling, PSU, and PSMU. A total of 428 adults (M_age_ = 40.82, SD = 12.54, range = 18 to 80) who were nearly equally represented in terms of sex (Male = 213, Female = 214, Prefer not to say = 1) from the United States were recruited to participate in the research by completing validated self-report measures. Structural Equation Modeling (SEM) was used to test two hypothesized models. Results indicated that binge-scrolling was positively associated with FoMO, PSU and PSMU and that the pathway between binge-scrolling and PSU/PSMU was mediated by FoMO. FoMO may amplify monitoring-related engagement, linking binge-scrolling to PSU and PSMU. Collectively, this research suggests that FoMO may function as a potential self-regulatory monitoring mechanism within compensatory technology use. Limitations and future research directions are discussed.

## 1. Introduction

Smartphones and social networking sites (SNS) are ubiquitous platforms in society. In the United States, 91% of individuals have a smartphone (Pew Research Center, 2025) and 73% use SNS (Baines, 2025). Both platforms are used frequently. In particular, Bradley and Howard (2023) found that the average daily smartphone use of young adults was 6 h and 53 min. They also measured average screentime per social media application, with TikTok use averaging 1 h and 55 min, Instagram use averaging 1 h and 26 min, and Snapchat averaging 1 h and 34 min. As smartphones are the main way individuals access social media (Wong, 2023), it is important to address how much of the screentime outlined earlier is likely due to the use of social media. The terms Problematic Smartphone Use (PSU) and Problematic Social Media Use (PSMU) have been proposed in the scholarly literature to describe excessive technology use that negatively impacts an individual’s life (Pérez-Torres, 2024; Wickord & Quaiser-Pohl, 2022). While much has been discovered about these concepts, the task of understanding factors associated with problematic technology use remains critical.

To further describe and study digital behaviors more specifically, the concept of binge-scrolling has been proposed. Binge-scrolling is described as consecutive viewing of digital content, such as short-form videos, photos, and SNS posts (Savci et al., 2025). Though like PSMU and PSU, binge-scrolling is different in the sense that while these other problematic behaviors are global to specific devices or environments, binge-scrolling pertains to a specific behavioral pattern that emerges within these environments and devices. Moreover, Savci et al. (2025) suggest that binge-scrolling should not be regarded as a problematic behavior, but rather a factor that contributes to the development of other problematic technology use behaviors. By conceptualizing specific aspects of technology use into terminology such as binge-scrolling, it facilitates a more granular examination of technology use.

Binge-scrolling has three unique factors: 1. Automatic scrolling: “habitual, repetitive scrolling behavior that occurs without a clear purpose” (Savci et al., 2025, p. 9), 2. Loss of control: “difficulties in resisting or regulating scrolling behavior” (Savci et al., 2025, p. 9), and 3. Negative outcomes: “adverse emotional and physical consequences associated with excessive scrolling” (Savci et al., 2025, p. 9). Empirical support for the automatic scrolling factor includes the finding that users consume more content and spend more time than intended when scrolling (Nong et al., 2023). This description highlights that when individuals engage with their devices or SNS platforms, they frequently scroll without a clear goal, often engaging in what can be described as mindless browsing. Loss of control differs from automatic scrolling in that the former reflects an inability to stop engaging in a behavior, whereas the latter is more strongly characterized by habitual and impulse-related tendencies. Moreover, loss of control represents an important dimension of binge-scrolling, as difficulties regulating behavior have been associated with both PSU (Billieux, 2012) and PSMU (Shannon et al., 2025), suggesting that binge-scrolling may conceptually relate with these problematic behaviors. Lastly, the negative outcomes dimension emphasizes how excessive scrolling behaviors may increase risk for adverse mental and physical health outcomes. This factor has support in the literature, as prior research has demonstrated that excessive scrolling is associated with depression (Qu et al., 2024), reduced attention (Park & Jung, 2024), deficits in cognitive functioning (Xu et al., 2025), and worsened sleep quality (Zhao & Kou, 2024)

Binge-scrolling shares theoretical and conceptual overlap with both PSU and PSMU; nevertheless, it has not been empirically examined whether binge-scrolling tendencies are associated with either of these constructs. Because binge-scrolling involves exposure to socially rewarding content, motivational processes pertinent to maintaining social connections may be of importance. Testing whether binge-scrolling is associated with both PSU and PSMU is important, as it may be linked with a greater risk of problematic technology use. Moreover, if these relationships exist, the mechanisms underlying these relationships remain unclear. A potential mediator is Fear of Missing Out (FoMO), which may explain how binge-scrolling is associated with PSU and PSMU. Namely, the extent to which an individual experiences concern about being absent from rewarding experiences may be associated with the link between binge-scrolling and PSU or PSMU.

The current study aims to understand the relationship between binge-scrolling, FoMO, and PSU/PSMU within the framework of Compensatory-Internet Use Theory (CIUT; Kardefelt-Winther, 2014). Based on extant theoretical and empirical findings, the proposed model is theoretically derived from CIUT and prior research linking binge-scrolling to FoMO (e.g., Zeba et al., 2026) and research linking FoMO to PSU (Elhai et al., 2025a) and PSMU (Elhai et al., 2025b). Specifically, we propose a model of behavior in which binge-scrolling, reflected as a higher-order construct with the three factors (e.g., automatic scrolling, loss of control, and negative outcomes), is an antecedent of FoMO, which in turn is associated with PSU/PSMU.

Binge-scrolling may occur in response to everyday stressors, such as negative affect or social exclusion, and can serve as a compensatory strategy to cope with these experiences or monitor socially relevant information, as reflected in FoMO. This behavior manifests across dimensions such as automatic scrolling, loss of control, and negative outcomes, which collectively capture the ways individuals engage with digital content. While these behaviors may initially serve a compensatory function, they can escalate into problematic technology use (PSU/PSMU), which is linked to negative outcomes including anxiety (Hou et al., 2023) and depression (Alavinikoo et al., 2025).

To further clarify these objectives of the study, the following research question is examined in this paper:

Research Question: How is binge-scrolling associated with problematic technology use, including Problematic Smartphone Use (PSU) and Problematic Social Media Use (PSMU), and what role does Fear of Missing Out (FoMO) play in this relationship?

This research question corresponds with hypotheses outlined in the sections below, which are tested simultaneously with the use of Structural Equation Modeling (SEM).

### 1.1. Theory: Compensatory-Internet Use Theory

Compensatory-Internet Use Theory (CIUT; Kardefelt-Winther, 2014) is a major theoretical perspective used to study problematic technology use. This perspective is rooted in the idea that technology use can function as a compensatory coping approach, in which individuals can use in a manner to cope with negative affect or unmet psychological needs, rather than due to inherently addictive properties of the technology itself (Kardefelt-Winther, 2014). A growing body of research has suggested how forms of negative affect including anxiety (Augner et al., 2023; Meng et al., 2025), depression (Augner et al., 2023), loneliness (Gao et al., 2025), and even suicidal ideation (Lee & Lee, 2023) are associated with digital engagement in online environments. Within the framework of CIUT, these increases in engagement are viewed as a maladaptive coping mechanism, rather than a behavioral addiction.

This theory is focused on the idea that psychological pathways can be influential in terms of how regular behavior ultimately becomes problematic. There are motivational factors that trigger smartphone use. For example, individuals with anxiety may use their smartphone to distract themselves from their anxious thoughts, manage their mood, or practice escapism motives by focusing on something external to them. Specifically, prior works have used this theoretical approach to describe both PSMU (Cui et al., 2023; Zhan et al., 2025) and PSU (Holte et al., 2024a; Liu et al., 2024; Wu et al., 2026), suggesting that CIUT provides a framework of examining how motivational processes like FoMO influence problematic behavior. Cui et al. (2023) reported a positive association between depression and PSMU. Similarly, with the use of Structural Equation Modeling, Zhan et al. (2025) found that digital stress, anxiety and depression each were associated with PSMU. Liu et al. (2024) examined the tenets of CIUT theory by testing a chain mediation model that depicted social anxiety and rumination as associations with PSU. Additional support comes from Wu et al. (2026), who found evidence for CIUT, demonstrating that rest intolerance, a negative feeling experienced when relaxing instead of working (Koo, 2023), was directly associated with both objective smartphone use and PSU. Lastly, Holte et al. (2024a) found that OCD symptoms are associated with PSU, which is consistent with the main theme of CIUT in that individuals use technology to cope with some form of negative affect. Taken together, these findings converge on the central premise of CIUT: that individuals engage in excessive or maladaptive technology use to regulate negative affective states.

In exploring the relationships between our variables of interest, we considered various theoretical frameworks. While the Stimulus-Organism-Response (SOR; Mehrabian & Russell, 1974) model is frequently used in the scholarly literature to examine how external stimuli trigger emotional and behavioral responses, it does not sufficiently capture the compensatory nature of technology use or the motivational processes underlying binge-scrolling. Thus, we chose to utilize CIUT, which emphasizes how individuals use technology as a coping mechanism to manage emotional distress or unmet psychological needs (Kardefelt-Winther, 2014). This theory is particularly relevant for understanding how binge-scrolling may be linked with problematic technology use.

### 1.2. Problematic Smartphone Use

Problematic Smartphone Use (PSU) has been conceptualized as excessive, compulsive, and uncontrollable smartphone use that involves a strong urge to check the smartphone, difficulty stopping, and interference with individuals’ daily functioning (Elhai et al., 2017). There is still an ongoing debate between addiction and process models, with some researchers suggesting that PSU is a behavioral addiction (Bragazzi & Del Puente, 2014; Chóliz, 2010), similar to other recognized addictions, while others propose that there are process-oriented explanations (Billieux et al., 2015; Panova & Carbonell, 2018). This debate is important for the current study as we evaluate the tenets of CIUT and how different mechanisms, such as motivational or regulatory pathways, influence the development of PSU. Within the perspective of CIUT, smartphones can function as compensatory tools in that they are mobile enough to be used across daily contexts (Holte et al., 2024b), which allows for constant management of one’s negative affect or psychological needs. Moreover, these aspects of smartphones allow users to monitor social information, with notifications and the use of various applications.

FoMO may facilitate PSU through the self-regulatory and social monitoring pathways consistent with CIUT. Specifically, FoMO might encourage users to use their smartphone to alleviate the concern of missing out on rewarding experiences (Holte, 2025). Similarly, FoMO may motivate users to monitor social information (Przybylski et al., 2013). Habitual smartphone engagement may be associated with PSU, with FoMO representing a potential pathway linking binge-scrolling and PSU. Consistent with compensatory use perspective, multiple sources suggest that the use of one’s smartphone can regulate affect and psychological needs (Holte et al., 2024a; Ng & Fam, 2024; Wu et al., 2026). While smartphones function as mobile compensatory tools, SNS platforms may represent environments where compensatory monitoring behaviors are amplified through platform-specific affordances.

The relationship between binge-scrolling and PSU is important to assess. We reason, through the frameworks of CIUT, that the immediate emotional relief individuals experience from scrolling through their smartphone encourages users to continue their behavior. Notably, prior work has highlighted how the affordances of smartphones and social media are specifically designed in a way to encourage habitual engagement (Chen et al., 2023). We argue that individuals who engage in binge-scrolling may be at greater risk for these technological habits which are, in turn, associated with PSU. Over time, this habitual use is associated with higher frequency and intensity of smartphone engagement, which can in itself be a form of negative affect. Through the lens of CIUT, this escalating use may be conceptualized as being associated with PSU. As a result, individuals find themselves struggling to regulate their smartphone usage and experience negative consequences, which are reflective of the characterization of PSU.

Taken together, we propose the following hypothesis:

**Hypothesis** **1:** *Binge-scrolling is positively associated with Problematic Smartphone Use.*

### 1.3. Problematic Social Media Use

Problematic Social Media Use (PSMU) has been described as excessive social media use that can cause negative behavioral outcomes (Pezzi et al., 2024). PSMU differs from PSU, where PSMU reflects platform-specific behavioral dysregulation, whereas PSU pertains to device-level dysregulation. This difference can be understood further when considering the specific affordances that social media platforms offer. For instance, social media platforms often feature infinite scroll feeds, such as algorithmically curated short-form video feeds. These affordances may create conditions that facilitate both FoMO and binge-scrolling behavior. Similarly, the affordance of users being able to post any content from their day-to-day experiences positions SNS platforms as environments for continuous social monitoring, which appears to be influential in terms of the development of FoMO (Holte & Ferraro, 2020).

By design, SNS expose users to continuous social information. Across most SNS platforms, user-created social content of text, image, or video format is densely embedded within platform interfaces. This exposure may facilitate both monitoring (Métellus et al., 2025) and comparison (Bazine et al., 2025) behaviors. Prior works have highlighted how both monitoring (Wang, 2025) and social comparison frequency (Piko et al., 2025) are associated with FoMO severity. It is plausible that FoMO may function as a self-regulatory monitoring mechanism associated with the link between binge-scrolling and PSMU. Within SNS environments, repeatedly monitoring social information may reinforce repetitive engagement patterns, increasing susceptibility to PSMU.

Like the reasoning underlying Hypothesis 1, which proposed that binge-scrolling would be positively associated with PSU, the same logic was extended to PSMU. In the present model, binge-scrolling is hypothesized to be associated with PSMU, as it may reflect a pattern of excessive and less regulated social media use. According to CIUT, individuals may turn to the Internet, including social media, to alleviate negative emotions or unmet psychological needs. The lack of intentionality in binge-scrolling undermines users’ ability to regulate their engagement, which may be associated with negative associations such as loneliness (Zeba et al., 2026), sleep deprivation (Zhang et al., 2023), and/or difficulties self-regulating (Zeba et al., 2026). From the perspective of CIUT, these negative outcomes may be compensated for through further social media use. When individuals experience emotional discomfort, they may use social media as a way to manage or distract them from these feelings. This creates a cycle of dependence where individuals could increasingly turn to social media to cope with the negative consequences of binge-scrolling. Over time, this compensatory behavior is associated with PSMU, where users may further lose control over their SNS use, and the consequences of this excessive use exacerbate the problem. As such, the following hypothesis is proposed:

**Hypothesis** **2:** *Binge-scrolling is positively associated with Problematic Social Media Use.*

### 1.4. Fear of Missing Out

Fear of Missing Out is conceptualized as “the apprehension that others might be having rewarding experiences from which one is absent” (Przybylski et al., 2013, p. 1841). As noted in Holte and Ferraro (2020), the rise of social media has amplified this experience, as individuals now see a curated depiction of the rewarding experiences and activities their friends attend, which would have otherwise gone unnoticed without the adoption of social media. Importantly, as it relates to CIUT, FoMO is more than a feeling, rather it also is a motivation to engage with social media, which is the key to the compensatory use cycle of CIUT. This motivation may manifest as a desire to stay up to date with rapidly evolving online trends, such as viral videos or popular social networking site (SNS) content. Supporting this notion, Zeba et al. (2026), found that individuals report binge-scrolling behaviors partially due to concerns about missing out on emerging norms and trends commonly disseminated through short-form digital content.

Additionally, FoMO has consistently been linked to concerns about social exclusion (Holte et al., 2022; Marengo et al., 2021), further supporting its relevance within compensatory use frameworks. Another important consideration is the very act of viewing SNS content itself, which may contribute to the development of FoMO. For example, prior work by David and Roberts (2023; Study 2) found that viewing SNS content, which is commonly accessed through smartphones, resulted in higher FoMO in contrast to viewing non-social media websites. Thus, repeated exposure to socially relevant digital content may heighten awareness of missed experiences and social comparison processes. Although FoMO is frequently conceptualized as a precursor to excessive technology use, the present study positioned binge-scrolling as an antecedent due to its role in prolonged exposure to socially relevant and rewarding digital content. Repeated exposure to such content may heighten perceptions of exclusion, social comparison, and awareness of missed experiences, thereby increasing FoMO severity. Collectively, these findings suggest a reciprocal but theoretically grounded relationship in which repeated exposure to socially relevant digital content, particularly through prolonged, passive consumptions patterns such as binge-scrolling, may contribute to heightened FoMO. Based on this reasoning and previous associations between binge-scrolling and FoMO (Zeba et al., 2026), we propose the following hypothesis:

**Hypothesis** **3:** *Binge-scrolling is positively associated with Fear of Missing Out.*

Drawing on the use of Self-Determination Theory (Deci & Ryan, 1985), the seminal work of Przybylski et al. (2013) suggested that individuals experience FoMO due to lack of psychological needs. From a lack of psychological needs, individuals experience FoMO, which is linked to technology engagement. For example, individuals cope with their uncertainty if they are missing out on a rewarding experience by checking their smartphone or social media feed, which is consistent with the tenets of CIUT in how individuals cope with negative affect through the use of technology use. Collectively, it is reasonable that FoMO is associated with both problematic smartphone (Elhai et al., 2025a) and social media use (Elhai et al., 2025b; Dadiotis & Roussos, 2024). Based on these previous findings, we proposed the following hypotheses:

**Hypothesis** **4:** *Fear of Missing Out is positively associated with Problematic Smartphone Use.*

**Hypothesis** **5:** *Fear of Missing Out is positively associated with Problematic Social Media Use.*

FoMO has been conceptualized as a behavioral correlate of these problematic behaviors and has also been examined as a mediator linking negative affect or unmet social needs with problematic technology use. Specifically, FoMO is proposed to translate basic levels of distress into the motivation of monitoring social information. With smartphones and social media being great platforms to monitor such information, technology use behaviors occur naturally as ways to alleviate any uncertainty an individual has and to maintain psychological needs satisfaction. Numerous works have applied FoMO as a mediator variable to explain the relationships between alexithymia and PSMU (Gori et al., 2024), anxiety and PSU (Elhai et al., 2020; Meng et al., 2025), obsessive–compulsive disorder severity and PSU (Holte et al., 2024a), attachment anxiety and smartphone attachment (Holte, 2025), intolerance of uncertainty and PSU (Yao et al., 2025), narcissism and PSMU (Giancola et al., 2025), and anxiety and social media fatigue (Świątek et al., 2021). Taken together, prior work provides increasing support for the role of FoMO as a mediator linking negative affect and deficits in psychological needs to compensatory technology engagement.

In our models, we propose that FoMO explains the relationship of binge-scrolling and PSU/PSMU. Specifically, we hypothesize that while binge-scrolling, individuals encounter social-related stimuli (e.g., posts about social events) that evoke a sense of FoMO. This feeling of missing out creates emotional discomfort, which individuals seek to alleviate, through the perspective of CIUT, by continuing to engage with social media or their smartphones. According to CIUT, this behavior is best understood as compensatory use, where individuals use technology to cope with negative emotions or unmet psychological needs. The emotional relief from continued scrolling and social media engagement helps individuals feel more connected, temporarily alleviating their feelings of FoMO. However, this pattern of compensatory use may be associated with more excessive and uncontrolled technology use, which is linked with PSU and PSMU. Therefore, FoMO may help account for the immediate emotional response associated with binge-scrolling and may serve as a mediator linking binge-scrolling and PSU/PSMU. Taken together, we propose the following hypotheses:

**Hypothesis** **6:** *Fear of Missing Out mediates the relationship of binge-scrolling and Problematic Smartphone Use.*

**Hypothesis** **7:** *Fear of Missing Out mediates the relationship between binge-scrolling and Problematic Social Media Use.*

### 1.5. Current Study

**Figure 1:**
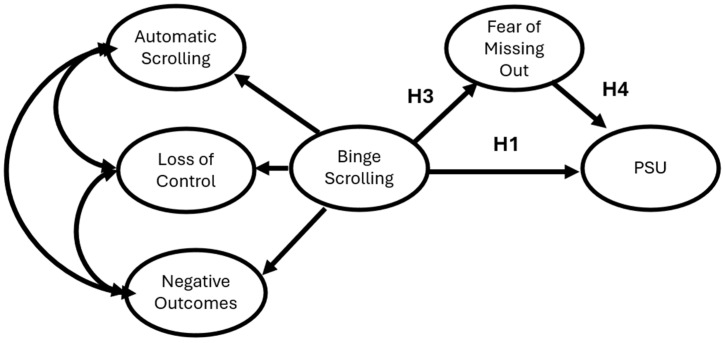
Hypothesized Structural Equation Model depicting binge-scrolling, Fear of Missing Out, and Problematic Smartphone Use.

Though much research has been conducted on the antecedents of PSU and PSMU, currently, there is a gap in the literature regarding whether binge-scrolling is associated with PSU or PSMU. By addressing this existing disparity, it may clarify how particular behavioral patterns relate to PSU and PSMU. Moreover, such research would address the recommendation of Modica and Bailey (2025), which suggested that researchers should shift their focus to specific types of smartphone use when applying the frameworks of CIUT. Within the current study, the specific type of smartphone use being studied is binge-scrolling. The aim of the current study is to test two theoretical Structural Equation Models to examine PSU and PSMU independently. As depicted in Figure 1, in our first model, binge-scrolling will be positively associated with both FoMO and PSU. Moreover, FoMO will be positively associated with PSU.

**Figure 2:**
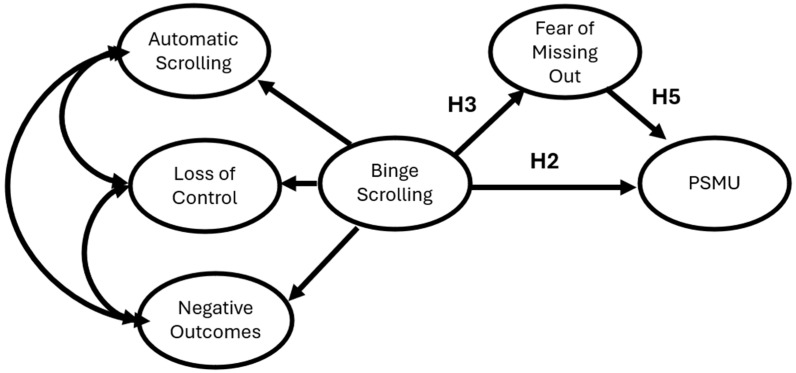
Hypothesized Structural Equation Model depicting binge-scrolling, Fear of Missing Out, and Problematic Social Media Use.

For the second model, as shown in Figure 2, binge-scrolling will be positively associated with both FoMO and PSMU. Like the first model, FoMO will be positively associated with PSMU in this model.

Based on CIUT, it is possible that individuals prone to binge scroll may be at risk for problematic technology use. In particular, individuals may become more dependent on social media or smartphone use for emotional regulation to counteract the unregulated engagement with these platforms as a function of binge-scrolling, and this reliance on smartphones and SNS use could be associated with compulsive use patterns. Through the perspective of CIUT, it is plausible that through binge-scrolling, individuals will come across content that triggers an increase in FoMO. This is based on previous research which suggests that viewing digital content can increase FoMO (David & Roberts, 2023; Study 2). Through the lens of CIUT, individuals may cope with negative affect, such as FoMO, through technology use (Kardefelt-Winther, 2014), which has been proposed to be associated with PSU and/or PSMU.

## 2. Methods

### 2.1. Participants

Participants initially included 451 individuals recruited through the CloudResearch Connect platform. Inclusion criteria included being a US adult with English fluency. Of this initial sample, 20 participants were excluded from further analyses for failing at least one of two instructed-response attention check items (e.g., “Please select ‘Moderately Disagree’ for this question” or “Please select ‘strongly agree’ for the question below”) and 3 were excluded due to leaving a qualitative attention check item (e.g., “What do you think this study was about.”) blank, suggesting they did not meet attention check criteria. The effective sample consisted of 428 adults (*M_age_* = 40.82, *SD* = 12.54, *range* = 18 to 80). For a detailed depiction of the sample, please refer to Table 1.

### 2.2. Materials

#### 2.2.1. Binge-Scrolling

The Binge-Scrolling Scale (Savci et al., 2025) is a questionnaire designed to measure binge-scrolling across three main dimensions: automatic scrolling, loss of control, and negative outcomes. This scale uses a 5-item Likert scale ranging from 1 “Never” to 5 “Always.” In total, each factor of this measure consists of four questions, for a total of 12 items. Example items include “I scroll continuously and without interruption” for automatic scrolling, “I scroll more than I planned” for loss of control, and “I feel physically uncomfortable after excessive scrolling” for negative outcomes. This measure displayed good internal consistency for automatic scrolling (*α* = 0.84), loss of control (*α* = 0.86), negative outcomes (*α* = 0.88), and the full scale (*α* = 0.90).

#### 2.2.2. Problematic Smartphone Use

We used the Smartphone Addiction Scale—Short Version (Kwon et al., 2013) to measure PSU. This unidimensional measure consists of 10 items and uses a Likert scale ranging from 1 “Strongly Disagree” to 6 “Strongly Agree” with no mid-point used. A representative item includes “I have a hard time concentrating in class, while doing assignments, or while working, due to smartphone use.” For our study, the measure displayed excellent internal consistency (*α* = 0.92).

#### 2.2.3. Problematic Social Media Use

The 9-item version of the Social Media Disorder Scale (SMDS; van den Eijnden et al., 2016) was used to measure PSMU. A Likert scale ranging from 1 “Never” to 5 “Very Often” was used to answer questions such as “During the past year, have you regularly felt dissatisfied because you wanted to spend more time on social media?” In this study, the measure demonstrated good internal consistency (*α* = 0.93).

#### 2.2.4. Fear of Missing Out

We used the 10-item Fear of Missing Out Scale (FoMOS; Przybylski et al., 2013) to measure Fear of Missing Out. Participants used a 5-item Likert scale ranging from 1 “Not at all true of me” to 5 “Extremely true of me”. An example item includes “I get anxious when I don’t know what my friends are up to.” For our study, we found good internal consistency with the FoMOS (*α* = 0.91).

#### 2.2.5. Demographics

Participants responded to four demographics questions about their sex, race/ethnicity, education, and geographic area of residence. A prescreen item that asked participants to report their age was completed right after participants consented to participate in the research.

#### 2.2.6. Filler Measures

To reduce demand characteristics and obscure the purpose of the study, several filler measures were included throughout the survey. Each of these measures were unrelated to the SEM model we examined and were intended to minimize hypothesis guessing and response bias. These measures included the Need to Belong Scale (NTBS; Leary et al., 2013), Life Orientation Test—Revised (Scheier et al., 1994), and the General Need Satisfaction Scale (Gagné, 2003).

### 2.3. Validity of Measures

#### 2.3.1. Discriminant Validity

To calculate discriminant validity, we applied the Fornell–Larcker criterion. We added covariance paths between each factor within each of the two models and recalculated the AVE based on its factor loadings. Next, we compared this value with the squared correlation value of each covariance path with other variables in the model. If the AVE for a factor is greater than the squared correlation with any other factor, the Fornell–Larcker criterion is met, providing evidence of discriminant validity. With the initial PSU model, evidence of discriminant validity was not supported with our measure of PSU and its relationship with the binge-scrolling factor, loss of control. Provided modifications to scales are permitted without a new sample to further validate; as long as the modification does not account for more than a 20% change in the items (Hair et al., 2010), limited item removal may be acceptable. Items 3 and 4 of the PSU measure were removed due to conceptual overlap with the loss of control factor of binge-scrolling, which may have contributed to inflated correlations and reduced discriminate validity. The 8-item PSU factor had excellent internal consistency (*α* = 0.92). Moreover, after removing items 3 and 4 from the PSU factor, evidence of discriminate validity was found across all factors of the first model. Similarly, evidence of discriminant validity was found with all factors of the PSMU model. A complete depiction of the Fornell–Larcker criterion results for Model 1 and the PSU model are shown in Table 2, while model 2, the PSMU model is depicted in Table 3.

#### 2.3.2. Convergent Validity

We assessed convergent validity of each factor prior to testing the full structural model. To examine convergent validity, we calculated the Average Variance Extracted (AVE) for each construct. AVE measures the extent to which each latent variable explains the variance in its indicators (e.g., items). In particular, it computes the average of the squared standardized factor loadings of the indicators for a specific construct. Common thresholds for good convergent validity are AVE values that exceed 0.50, as this indicates the latent variable explains more than half of the variance of its indicators. To provide clarity and provide adequate precision, AVE values were rounded to three decimal places. Below in Table 4 you will find the AVE for each construct.

### 2.4. Procedure

Prior to data collection, the Institutional Review Board approved all procedures and protocol related to the study (Protocol Submission Number: IRB-26-AH-229). All participants in the research were recruited from CloudResearch’s Connect platform, which uses a multi-layered system to promote the acquisition of quality data. For this research, each participant received $1.00 for their time. Data collection took place on 2 February 2026. This study was hosted on Qualtrics survey platform, which allowed for the questionnaires listed above to be presented randomly, thus diminishing the likelihood that order effects occurred. Similarly, the prevent ballot-stuffing option was selected within the Qualtrics survey, which prevented participants from taking the research study more than once. All measures were equipped within their own Qualtrics block and were randomized at the block level. One of the attention checks was placed in the demographics block and the other was placed in the middle of the NTBS block. On average, the procedure took participants 10.93 (*SD* = 7.20) minutes.

### 2.5. Analysis

Both IBM SPSS Statistics (Version 30) and IBM SPSS AMOS Structural Equation Modeling Software (Version 26) were used to analyze our data. Namely, SPSS was used to conduct descriptive and correlational statistics and AMOS was used to test our hypothesized Structural Equation Models. As the Qualtrics survey was designed to remind participants of any missing items, no missing data were present. We used Confirmatory Factor Analysis (CFA) with the maximum likelihood technique on each SEM model, which was used to test our main hypotheses. Model fit was evaluated using commonly reported SEM indices, including comparative fit index (CFI), root-mean-square error of approximation (RMSEA), Tucker Lewis Index (TLI), and standardized root mean square residual (SRMR). We used established guides to evaluate our model. In particular, CFI and TLI values of 0.90 or higher would be considered acceptable (Byrne, 1994; Marsh et al., 2004a), SRMR values below or equal to 0.08 would be good (Hu & Bentler, 1999), and RMSEA values less than or equal to 0.08 (MacCallum & Hong, 1997) would be acceptable fit. Model respecifications were conducted conservatively and only when theoretically justifiable. Covariance paths were added conservatively between error terms of items within the same factor when modification indices were 25 or greater and the additions were theoretically justifiable (Marsh et al., 2004b). Reference to the item pairs (e.g., FoMOS 1–2) indicate correlated residuals between those specific scale items.

Prior to evaluating the structural fit of our models, we used the Harman single-factor test (Harman, 1976) to examine if common bias was influencing the results in the data. With this test, all items that make up the factors of each individual SEM model were constrained to one factor in Exploratory Factor Analysis by way of Principal Axis Factoring. If the shared variance did not exceed 50% threshold, it would suggest that common bias was not present in the data. Additionally, following the advice of Kock (2015), we examined the full collinearity variance inflation factors (VIFs) and compared the values obtained to the recommended values of 3.3. Next, we tested our two main SEM models. Binge-scrolling was modeled as a higher-order latent construct reflected by three lower-ordered factors: automatic scrolling, loss of control, and negative outcomes. The lower-order factors demonstrated sufficient shared variance to justify their inclusion within a higher-order binge-scrolling construct. Structural paths were then estimated from the higher-order binge-scrolling latent variable to FoMO and PSU (Model 1) and PSMU (Model 2). In addition, FoMO was expected to be positively associated with PSU (Model 1) and PSMU (Model 2). Lastly, FoMO was anticipated to mediate the relationship between binge-scrolling and PSU (Study 1) and PSMU (Study 2). Our mediational analyses were powered by 5000 bootstrapped samples and a 95% confidence interval. In addition, we calculated R^2^ for each endogenous variable (e.g., FoMO, PSU, and PSMU) to detect how much variance of the variable is explained by predictor variables. After testing our initial hypothesized models, we tested two alternative models, featuring the variables in reverse order.

## 3. Results

Preliminary results indicated that the skewness (−0.57 to 2.04) and kurtosis values (−1.31 to 3.08) fell within the recommended thresholds made by Brown (2006), having kurtosis values between −10 to 10 and skewness between −3 and 3. Table 5 outlines a depiction of both descriptive and correlational statistics. Results of the Harman single-factor test for variables within the first model suggest that the shared variance of all items was 39.49%, which is less than the threshold of 50%. Similarly, the Harman single-factor test for the second model variables had shared variance at 40.00%, which is less than the 50% threshold. Similarly, we used Kock’s (2015) full collinearity VIF approach and found that the highest VIF value for the PSU model factors was 3.08 and the highest value for the PSMU model factors was 3.01. All VIF values fell below the recommended threshold of 3.3, suggesting common method bias was unlikely.

Provided the original SMDS used binary scoring (e.g., 1 for “yes” and 0 for “no”), we conducted a CFA to affirm the psychometric properties of the measure in the context of having five Likert options in lieu of two. After applying three modification paths between error terms of individual items, the measure displayed excellent psychometric fit (TLI = 0.97, CFI = 0.98, SRMR = 0.04), though the RMSEA value (0.09) was a bit higher than conventional thresholds. It is important to note that, as outlined by Kenny et al. (2015), the reliability of RMSEA is reduced in models with very few degrees of freedom. Because the present model had low degrees of freedom, the RMSEA value may have been inflated; therefore, greater emphasis was placed on the TLI, CFI, and SRMR.

### 3.1. Model 1 Structural Equation Modeling Results

**Figure 3:**
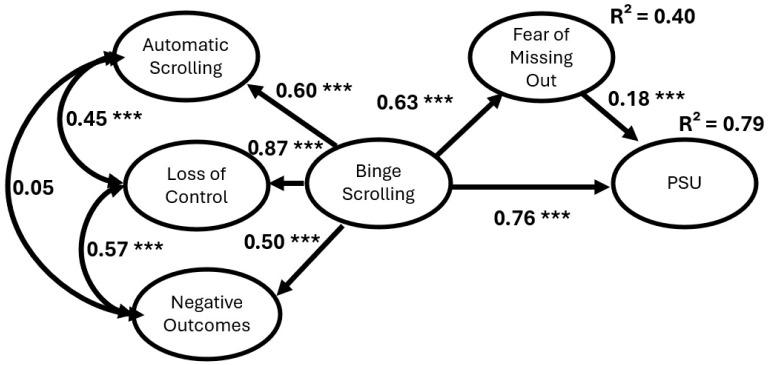
Structural Equation Modeling with factor loadings depicting binge-scrolling, FoMO and Problematic Smartphone Use with factor loadings. Note: *** *p* < 0.001; PSU—Problematic Smartphone Use.

Our initial fit for our first iteration of Model 1 demonstrated poor fit in the following indices of CFI (0.83), TLI (0.82) and RMSEA (0.09), though it displayed acceptable fit with SRMR (0.07). Three theoretically justified covariance paths were added to improve model fit (see Table 6). After these covariance paths were added, our model had acceptable fit with CFI (0.91), RMSEA (0.07), and SRMR (0.07), and TLI (0.90). As shown in Figure 3, all paths, with the exception of the covariance path between automatic scrolling and negative outcomes (*p* = 0.48), were statistically significant. The lower-order binge-scrolling dimensions demonstrated sufficient shared variance to support the higher-order latent construct. Accordingly, the reported β coefficients reflect structural paths estimated from the higher-order binge-scrolling construct rather than from the individual lower-order dimensions independently. In addition, it was revealed that binge-scrolling was positively associated with FoMO and PSU, FoMO was positively associated with PSU, and FoMO mediated the relationship between binge-scrolling and PSU (β = 0.11, S.E. = 0.05, *p* = 0.006, 95% CI = 0.06 to 0.25). Lastly, FoMO (R^2^ = 0.40) and PSU (R^2^ = 0.79) were both substantially explained by the model.

### 3.2. Model 2 Structural Equation Modeling Results

**Figure 4:**
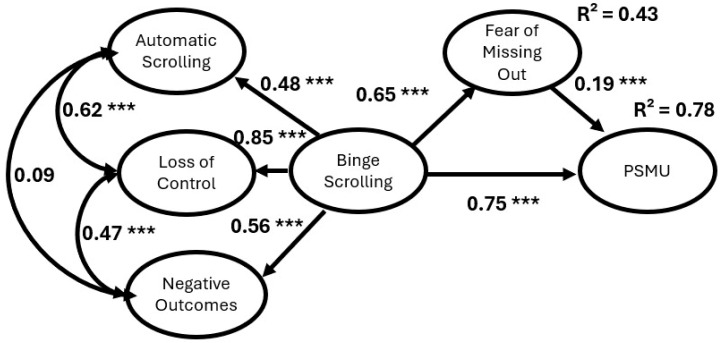
Structural Equation Modeling with factor loadings depicting Binge-scrolling, FoMO and Problematic Social Media Use with Factor Loadings. Note: *** *p* < 0.001; PSMU—Problematic Social Media Use.

The initial fit for Model 2 was acceptable with the SRMR index (0.08), though TLI (0.81), CFI (0.82), and RMSEA (0.10) left room for improvement. Six covariance paths were added to improve model fit (see Table 7). After applying these covariance paths, model fit was improved. In particular, the indices of TLI (0.90), CFI (0.91), RMSEA (0.07) and SRMR (0.08) were all within the acceptable conventional cutoff values. Consistent with Model 1, the reported β coefficients reflect structural paths estimated from the higher-order binge-scrolling construct. As shown in Figure 4, all pathways, with the exception of the covariance path of automatic scrolling and negative outcomes (*p* = 0.17), were statistically significant at the 0.001 level. Similarly, binge-scrolling was positively associated with FoMO and PSMU. FoMO was positively associated with PSMU and significantly mediated the relationship between binge-scrolling and PSMU (β = 0.12, S.E. = 0.04, *p* = 0.001, 95% CI = 0.06 to 0.19). Lastly, FoMO (R^2^ = 0.43) and PSMU (R^2^ = 0.78) were both substantially explained by the model.

### 3.3. Alternative Models

To examine the plausibility of alternative directional relationships among the study variables, we tested two additional SEM models that reversed the ordering of the focal constructs. Specifically, Models 3 and 4 evaluated whether PSU and PSMU could instead function as antecedents associated with FoMO and binge-scrolling, rather than as outcomes of these variables. In both alternative models, FoMO was specified as an intermediary variable, with binge-scrolling modeled as an endogenous construct. In both models, the higher-order factor model of the binge-scrolling factors (e.g., automatic scrolling, loss of control, and negative outcomes) failed to converge without producing an inadmissible parameter estimate (i.e., a Heywood case with a standardized loading exceeding 1). This may suggest potential issues with the assumed hierarchical structure. The presence of the Heywood case may indicate potential model misspecification, estimation instability, or problems with the latent structure. As improper solutions can produce bias or uninterpretable parameter estimates, we did not retain Models 3 and 4 for further consideration. Moreover, because Models 1 and 2 included a theoretically derived second-order factor structure that was absent in Models 3 and 4, direct comparisons across these models would conflate differences in model fit with differences in model specification. Therefore, model comparisons between were restricted to admissible models consistent with the underlying theoretical framework.

## 4. Discussion

This paper aimed to develop an understanding of the mechanisms of how behavior is associated with problematic technology use. Through the lens of CIUT, we evaluated how binge-scrolling tendencies are associated with Problematic Smartphone and Problematic Social Media Use through the pathway of Fear of Missing Out. Collectively, results indicate that FoMO functions as a motivational pathway and a self-regulatory monitoring mechanism used to compensate for the uncertainty about missing rewarding experiences, which in turn may be associated with problematic technology use patterns. We found support for each of our hypotheses. Results indicated that binge-scrolling was positively associated with PSU (Hypothesis 1) and PSMU (Hypothesis 2). This may indicate that frequent scrolling behaviors are associated with problematic technology use. One reason for this is that both these technological affordances may encourage habitual engagement (Chen et al., 2023). Thus, users who binge scroll may be more likely to report problematic technology use behaviors. Across both models, binge-scrolling was positively associated with FoMO (Hypothesis 3). Given that prior work found that scrolling on SNS platforms can result in higher FoMO severity (David & Roberts, 2023; Study 2), this result is consistent with prior findings. It was also revealed that FoMO was positively associated with PSU (Hypothesis 4) and PSMU (Hypothesis 5). Given that prior work has established that FoMO is an antecedent to PSU (Elhai et al., 2025a) and PSMU (Elhai et al., 2025b), this is reasonable. Across both models, FoMO mediated the relationship between binge-scrolling and PSU (Hypothesis 6) and PSMU (Hypothesis 7). This finding is consistent with the tenets of CIUT. In this example, binge-scrolling is associated with PSU and PSMU by motivating users to monitor social information (Przybylski et al., 2013).

The results of this study can be conceptualized by viewing binge-scrolling as potentially a behavioral feature linked to compensatory technology use. Namely, our study suggests that specific scrolling patterns may matter in relation to PSU and PSMU. One implication of these findings is that it may be related to these problematic behaviors. As noted earlier, by studying specific user behaviors, we can have a more granular understanding of the individual differences in such technological uses and how these uses can become problematic. This followed the recommendation of Modica and Bailey (2025), which suggested that future smartphone research should study specific smartphone use behaviors. Binge-scrolling itself may be a way to cope with negative forms of affect, such as anxiety or depression, and future research may further examine such understanding.

The role of FoMO in this study’s results aligns well with the extant literature. Specifically, Przybylski et al. (2013) argued that individuals with FoMO are likely to track social updates to evaluate their concern they may be missing out. In this way, FoMO may function as an active monitoring tool used by individuals to screen social information to maintain or restore psychological need satisfaction. As it relates to the current study, users are actively regulating their uncertainty through scrolling, with this regulation reflecting information-seeking, social monitoring, and self-regulation. It would appear based on our results that FoMO may explain how scrolling behaviors become associated with problematic technology use.

In addition to understanding the role of FoMO in the relationship between binge-scrolling and problematic technology use, it is important to emphasize the implications of scrolling behaviors being linked to FoMO. These findings extend prior work by suggesting that scrolling behaviors may be associated with higher levels of FoMO. Prior work found that browsing social media for 15 min caused individuals to have more FoMO than browsing non-social media websites (David & Roberts, 2023; Study 2). In addition, the work of Groenestein et al. (2024) found that social media use and FoMO have a cyclical relationship where increases in FoMO cause more SNS use, and more SNS use is associated with FoMO, suggesting a reinforcement feedback loop. Taken together, the present findings suggest that SNS and smartphone use may not only be active in areas of monitoring and compensatory regulation but could also be associated with higher FoMO.

Moreover, with this being one of the earliest papers to use the three-factor Binge-Scrolling Scale, it is important to consider the behavior of each factor within the structural models. At the bivariate level, all three factors, automatic scrolling, loss of control, and negative outcomes, were significantly and positively correlated, suggesting initial coherence of the construct. However, within the SEM framework, automatic scrolling was not significantly associated with negative outcomes when accounting for the shared variance among factors. This finding raises an important conceptual consideration. Although these factors are theorized to reflect related components of binge-scrolling, their relationships may differ once modeled simultaneously. One possible interpretation is that automatic scrolling may reflect a more habitual or passive form of engagement that does not necessarily translate into negative outcomes unless accompanied by a loss of control. Future research is needed to determine whether this pattern reflects a stable distinction between components of binge-scrolling or is sample-specific. In contrast, the loss of control factor demonstrated consistently strong loadings (e.g., 0.87 for the PSU model and 0.85 for the PSMU model), suggesting that the perceived inability to regulate use may be a pivotal aspect of binge-scrolling. Prior work by Park and Jung (2024) highlighted how the role of device or platform affordances, such as infinite scrolling, contributed to this diminished sense of control. However, further research is needed to examine individual differences that may make some people more susceptible to this aspect of binge-scrolling.

In terms of practical implications, the present study contributes to a growing awareness of how binge-scrolling may be linked to PSU and PSMU. While causal research is still needed, these findings provide an important first step for clinicians and researchers in recognizing that individuals who frequently engage in binge-scrolling may be at greater risk for problematic technology use behaviors. An important next step in this line of research is to examine the temporal ordering of the three binge-scrolling factors identified by Savci et al. (2025). Clarifying how these dimensions relate to one another, and whether certain factors are more strongly associated with negative outcomes, may help inform the timing and focus of targeted interventions. Beyond testing temporal ordering, identifying additional factors associated with binge-scrolling should remain a priority, as such efforts may support the development of more precise and timely intervention strategies.

Taken together, one conceptual implication of the present study is the concept that PSU and PSMU may be better studied as an ongoing regulatory process, rather than solely as a fixed behavioral addiction. This suggestion reinforces the main tenets of CIUT. Namely, the pathways of binge-scrolling to FoMO to PSU/PSMU reflect the dynamic nature of how individuals use technology to self-regulate, and through this process, users may be associated with these habitual behavioral patterns. Another important implication is that binge-scrolling may serve as an early behavioral indicator of behaviors that are associated with the risk of becoming problematic. Future research should explore methods to alert individuals of these micro-behavioral patterns, which may make users more prone to PSU or PSMU. Lastly, though CIUT is often studied through the lens of escapism, by incorporating FoMO into this model, we propose that monitoring social information may be an additional compensatory pathway. While the act of monitoring social information may distract the user from their negative affect or lack of psychological needs, we reason that in the case of FoMO, it is an important step for users when they actively seek an answer to the question “am I missing out?”

### Limitations

Aside from the strengths of this paper, including using advanced statistical methods, several limitations should be acknowledged. First, this study used a cross-sectional, non-experimental research design. In other words, our proposed models reflect theoretically informed directional hypotheses, rather than a confirmed causal sequence. This means that this study’s design limits the ability to make causal inference and temporal interpretation. Similarly, though the present set of models were grounded in Compensatory-Internet Use Theory (Kardefelt-Winther, 2014), the cross-sectional nature of the data precludes definitive conclusions regarding the causal directionality of the relationships examined. Although the current study conceptualized binge-scrolling as an antecedent to FoMO, the cross-sectional nature of the data prevents conclusions regarding temporal ordering or causality. It is equally possible that individuals high in FoMO may engage in greater binge-scrolling behaviors. Future longitudinal and experimental research should examine the potential bidirectional relationship between these constructs. More broadly, reverse causation may be possible, such that individuals with higher PSU or PSMU may experience more FoMO and thus have more binge-scrolling tendencies. The results of the current study do not dismiss this possibility, and future work is encouraged to test the causal influence of these variables to have a better understanding of how they interact with each other. Likewise, it is plausible that these relationships may be influenced by third variables that we did not consider, such as trait anxiety, reward sensitivity, or broader addictive processes. Future research could expand our findings by applying our research in such a way that allows for longitudinal effects to be examined, such as the work of Groenestein et al. (2024), who found that FoMO at Time 1 was positively associated with SNS use at Time 2, which in turn was positively associated with FoMO at Time 3. Alternatively, studying this topic with experimental approaches will also allow researchers to study these constructs in a manner that allows for causal claims to be made about the topic.

Another limitation of the present study is that, although SEM models were consistent with an association in which binge-scrolling was linked to FoMO, which is in turn linked to PSU/PSMU, the model did not include factors that may help explain why individuals engage in binge-scrolling initially. Drawing on CIUT, it is plausible that unmet psychological needs or negative affect may be associated with binge-scrolling, which may in turn relate to greater FOMO-related cognitions and compensatory technology use patterns linked to PSU/PSMU. However, these interpretations remain speculative, and future research is needed to examine these relationships directly. In addition, we modified the response format of the PSMU measure to allow for more fine-grained assessment. Specifically, we changed the scoring from a dichotomous response format to a Likert format that provided the participants with five options to choose from. While this modification may provide greater sensitivity in capturing variability in behavior, future research is needed to evaluate the construct validity of the measure under this revised scoring format.

It is important to address that participants recruited from platforms such as Mechanical Turk, which is similar to CloudResearch, have historically shown to score higher in variables pertaining to anxiety and depression, in contrast to samples recruited through traditional recruitment approaches (Arditte et al., 2016), which may limit representativeness. In addition, our sample was primarily White, and future research should explore the findings in a more diverse sample. As a result, our study had a restriction of range in demographic variables, limiting the representation of the broader target population. Consequently, our findings may not fully generalize to more diverse demographic groups. This is particularly noted given prior work (Debb et al., 2022) which has identified differences in the factor structure of the FoMOS across racial groups. Similarly, the present sample only consisted of English-speaking adults, which could question the generalizability of the findings in non-English speaking populations or individuals from different cultures. In addition, the amount of money awarded for participation (e.g., $1.00) may have been a stronger motivator for some individuals than for others, potentially under-representing participants who consider the financial compensation insufficient for their time. On a different note, like any study that uses self-report measures, there will always be the concern of social desirability responses. Participants could have provided responses that were either under or over reported to be perceived as desirable, which could have influenced the results of the observed relationships in our study. Although steps such as anonymity were implemented to reduce this risk, future research may benefit from using multi-method approaches (e.g., behavioral or passive data collection) to better account for this potential bias.

## 5. Conclusions

Aside from these limitations, this research provides initial evidence suggesting binge-scrolling may be an early behavioral indicator of behaviors that are associated with problematic technology use. This finding may inform prevention, psychoeducation, digital well-being interventions, or early identification of problematic technology use patterns in everyday contexts. Future research may build upon this work by developing guidelines and best practices to identify which specific dimensions of binge-scrolling are more strongly linked to problematic technology use. Our mediational results suggest that FoMO is a self-regulatory monitoring mechanism which helps explain the pathway of how binge-scrolling may be linked with either PSU or PSMU. Lastly, our findings are consistent with the major tenets of CIUT, which suggest that PSU and PSMU are both dynamic processes and may offer a complementary perspective to the addictions-based frameworks. Taken together, this work contributes an awareness of why it is important to study specific smartphone use behaviors from an approach that allows for a more granular observation. By adopting this more granular approach, future work may more systematically investigate how specific technological behaviors across SNS and smartphones shape human engagement.

## Figures and Tables

**Table 1:** Demographic statistics.

Variable	*n*	%
Sex		
Male	213	49.77%
Female	214	50.00%
Prefer Not to Disclose	1	0.23%
Race/Ethnicity		
White	309	72.20%
Black or African American	64	14.95%
American Indian or Alaska Native	5	1.17%
Asian	43	10.05%
Native Hawaiian or Pacific Islander	2	0.47%
Hispanic or Latino	25	5.84%
Middle Easterner	2	0.47%
Lebanese	1	0.23%
Other—Did Not Indicate	4	0.94%
Highest Education Degree Obtained		
High School/GED	108	25.23%
Associates	54	12.62%
Bachelor’s	188	43.93%
Master’s	66	15.42%
Doctorate	12	2.80%
Geographic Area of Residence		
Southeast	121	28.27%
Northeast	95	22.20%
West	91	21.26%
Midwest	67	15.65%
Southwest	53	12.38%
Did Not Respond to Question	1	0.23%

Note: As individuals are able to endorse multiple races/ethnicities, the summation of this category exceeds 100%.

**Table 2:** Results of Fornell–Larcker criterion.

Construct	AVE	Squared Correlation with Factor 1	Squared Correlation with Factor 2	Squared Correlation with Factor 3	Squared Correlation with Factor 4	Squared Correlation with Factor 5	Pass Fornell–Larcker Criterion?
1. BS–AS	0.568	-	0.490	0.116	0.144	0.281	Yes
2. BS–LOC	0.623	0.490	-	0.462	0.303	0.578	Yes
3. BS–NO	0.646	0.116	0.462	-	0.176	0.185	Yes
4. FoMO	0.502	0.144	0.303	0.176	-	0.423	Yes
5. PSU	0.596	0.281	0.578	0.185	0.423	-	Yes

Notes: AVE—Average Variance Extracted; BS—Binge-Scrolling; AS—Automatic Scrolling; LOC—Loss of Control; NO—Negative Outcomes; FoMO—Fear of Missing Out; PSU—Problematic Smartphone Use. AVE values here are estimated as part of the full SEM model.

**Table 3:** Results of Fornell-Larcker criterion.

Construct	AVE	Squared Correlation with Factor 1	Squared Correlation with Factor 2	Squared Correlation with Factor 3	Squared Correlation with Factor 4	Squared Correlation with Factor 5	Pass Fornell–Larcker Criterion?
1. BS–AS	0.568	-	0.490	0.116	0.144	0.137	Yes
2. BS–LOC	0.627	0.490	-	0.462	0.303	0.504	Yes
3. BS–NO	0.646	0.116	0.462	-	0.176	0.230	Yes
4. FoMO	0.502	0.144	0.303	0.176	-	0.422	Yes
5. PSMU	0.626	0.137	0.504	0.230	0.422	-	Yes

Notes: AVE—Average Variance Extracted; BS—Binge-Scrolling; AS—Automatic Scrolling; LOC—Loss of Control; NO—Negative Outcomes; FoMO—Fear of Missing Out; PSMU—Problematic Social Media Use. AVE values here are estimated as part of the full SEM model.

**Table 4:** Average Variance Extracted for factors within the models.

Construct	AVE
Binge-scrolling—Automatic Scrolling Factor	0.565
Binge-scrolling—Loss of Control Factor	0.624
Binge-scrolling—Negative Outcomes Factor	0.646
Fear of Missing Out	0.503
Problematic Smartphone Use	0.593
Problematic Social Media Use	0.626

Note: AVE = Average Variance Extracted. AVE values in this table are calculated directly from the items of each individual scale. Slight differences exist between these values and the AVE reported in Table 2 and Table 3 because the Fornell–Larcker criterion tables estimate AVE with the full SEM model, which accounts for all factors and covariances simultaneously.

**Table 5:** Descriptive and correlational statistics.

	Mean	SD	Range	2	3	4	5	6	7
1. BS–AS	11.79	3.56	4–20	0.62 ***	0.29 ***	0.76 ***	0.34 ***	0.40 ***	0.49 ***
2. BS–LOC	8.93	3.58	4–20		0.59 ***	0.89 ***	0.49 ***	0.68 ***	0.70 ***
3. BS–NO	9.09	4.05	4–20			0.79 ***	0.37 ***	0.46 ***	0.40 ***
4. BS–Total	29.80	9.10	12–59				0.49 ***	0.63 ***	0.64 ***
5. FoMO	22.39	8.74	10–46					0.60 ***	0.61 ***
6. PSMU	16.74	7.69	9–45						0.82 ***
7. PSU	19.64	9.27	10–46						

Notes: BS—Binge-scrolling; AS—Automatic Scrolling; LOC—Loss of Control; NO—Negative Outcomes; FoMO—Fear of Missing Out; PSMU—Problematic Social Media Use; PSU—Problematic Smartphone Use; *** *p* < 0.001.

**Table 6:** Fit indices of each iteration of the Problematic Smartphone Use Structural Equation Model after the addition of covariance paths on error terms.

Path Added	MI	CFI	TLI	SRMR	RMSEA	RMSEA 90% CI
None	None	0.84	0.83	0.07	0.09	0.086–0.094
FoMOS 1–2	272.19	0.88	0.87	0.07	0.08	0.073–0.081
FoMOS 7–9	129.41	0.90	0.89	0.07	0.07	0.067–0.076
SAS-SV 1–2	62.46	0.91	0.90	0.07	0.07	0.064–0.073

Notes: All added covariance paths were statistically significant (*p* < 0.001); MI—Modification Index; FoMOS—Fear of Missing Out Scale; SAS-SV—Smartphone Addiction Scale—Short Version.

**Table 7:** Fit indices of each iteration of the Problematic Social Media Use Structural Equation Model after the addition of covariance paths on error terms.

Path Added	MI	CFI	TLI	SRMR	RMSEA	RMSEA 90% CI
None	None	0.82	0.81	0.08	0.10	0.09–0.10
FoMOS 1–2	274.27	0.86	0.85	0.08	0.09	0.08–0.09
FoMOS 7–9	131.54	0.88	0.86	0.08	0.08	0.08–0.09
SMDS 7–9	119.98	0.89	0.88	0.08	0.08	0.07–0.08
SMDS 6–9	52.71	0.90	0.89	0.08	0.08	0.07–0.08
SMDS 6–7	45.94	0.91	0.90	0.08	0.07	0.07–0.08
SMDS 5–8	26.83	0.91	0.90	0.08	0.07	0.07–0.08

Notes: All added covariance paths were statistically significant (*p* < 0.001); MI—Modification Index; FoMOS—Fear of Missing Out Scale; SMDS—Social Media Disorder Scale.

## Data Availability

Data will be made available on reasonable request.

## References

[B1-behavsci-16-00802] Alavinikoo S., Elhai J. D., Hutcheson E. F., Montag C. (2025). Underlying dimensions of depression and negative affect are equally related to problematic smartphone and social media use severity. Psychiatry Research.

[B2-behavsci-16-00802] Arditte K. A., Çek D., Shaw A. M., Timpano K. R. (2016). The importance of assessing clinical phenomena in Mechanical Turk research. Psychological Assessment.

[B3-behavsci-16-00802] Augner C., Vlasak T., Aichhorn W., Barth A. (2023). The association between problematic smartphone use and symptoms of anxiety and depression—A meta-analysis. Journal of Public Health.

[B4-behavsci-16-00802] Baines G. (2025). What percentage of Americans use social media?.

[B5-behavsci-16-00802] Bazine N., Serra J., Giunchi M., Peña-Jimenez M. (2025). The negative consequences of networking through social network services: A social comparison perspective. Computers in Human Behavior.

[B6-behavsci-16-00802] Billieux J. (2012). Problematic use of the mobile phone: A literature review and a pathways model. Current Psychiatry Reviews.

[B7-behavsci-16-00802] Billieux J., Maurage P., Lopez-Fernandez O., Kuss D. J., Griffiths M. D. (2015). Can disordered mobile phone use be considered a behavioral addiction? An update on current evidence and a comprehensive model for future research. Current Addiction Reports.

[B8-behavsci-16-00802] Bradley A. H. M., Howard A. L. (2023). Stress and mood associations with smartphone use in university students: A 12-Week Longitudinal Study. Clinical Psychological Science.

[B9-behavsci-16-00802] Bragazzi N. L., Del Puente G. (2014). A proposal for including nomophobia in the new DSM-V. Psychology Research and Behavior Management.

[B10-behavsci-16-00802] Brown T. A. (2006). Confirmatory factor analysis for applied research.

[B11-behavsci-16-00802] Byrne B. M. (1994). Burnout: Testing for validity, replication, and invariance of causal structure across elementary, intermediate, and secondary teachers. American Educational Research Journal.

[B12-behavsci-16-00802] Chen X., Hedman A., Distler V., Koenig V. (2023). Do persuasive designs make smartphones more addictive?—A mixed-methods study on Chinese university students. Computers in Human Behavior Reports.

[B13-behavsci-16-00802] Chóliz M. (2010). Mobile phone addiction: A point of issue. Addiction.

[B14-behavsci-16-00802] Cui J., Wang Y., Liu D., Yang H. (2023). Depression and stress are associated with latent profiles of problematic social media use among college students. Frontiers in Psychiatry.

[B15-behavsci-16-00802] Dadiotis A., Roussos P. (2024). Relationship between FoMO, problematic social media use, self-esteem, negative affectivity, and physical exercise: A structural equation model. Journal of Technology in Behavioral Science.

[B16-behavsci-16-00802] David M. E., Roberts J. A. (2023). The dual nature of social media: Examining the direction of causal flow between fear of missing out and social media use. Cyberpsychology, Behavior, and Social Networking.

[B17-behavsci-16-00802] Debb S. M., Haschke K. J., McClellan M. K. (2022). Validation of the fear of missing out scale for use with African Americans in the United States. Cyberpsychology, Behavior, and Social Networking.

[B18-behavsci-16-00802] Deci E. L., Ryan R. M. (1985). Intrinsic motivation and self-determination in human behavior.

[B19-behavsci-16-00802] Elhai J. D., Casale S., Bond R. A. (2025a). FoMO’s apprehension of missing out and constant connection desire dimensions differentially correlate with problematic smartphone and social media use, but not with depression or generalized anxiety. Journal of Anxiety Disorders.

[B20-behavsci-16-00802] Elhai J. D., Casale S., Montag C. (2025b). Worry and fear of missing out are associated with problematic smartphone and social media use severity. Journal of Affective Disorders.

[B21-behavsci-16-00802] Elhai J. D., Dvorak R. D., Levine J. C., Hall B. J. (2017). Problematic smartphone use: A conceptual overview and systematic review of relations with anxiety and depression psychopathology. Journal of Affective Disorders.

[B22-behavsci-16-00802] Elhai J. D., Yang H., Fang J., Bai X., Hall B. J. (2020). Depression and anxiety symptoms are related to problematic smartphone use severity in Chinese young adults: Fear of missing out as a mediator. Addictive Behaviors.

[B23-behavsci-16-00802] Gagné M. (2003). The role of autonomy support and autonomy orientation in prosocial behavior engagement. Motivation and Emotion.

[B24-behavsci-16-00802] Gao J., Xu D., Romano D., Hu X. (2025). Acculturative stress, loneliness, smartphone addiction, L2 emotions, and creativity among international students in China: A Structural equation model. Frontiers in Psychiatry.

[B25-behavsci-16-00802] Giancola M., Perazzini M., Bontempo D., Perilli E., D’Amico S. (2025). Narcissism and Problematic Social Media Use: A moderated mediation analysis of Fear of Missing Out and trait mindfulness in youth. International Journal of Human-Computer Interaction.

[B26-behavsci-16-00802] Gori A., Topino E., Gioia F., Casale S. (2024). Problematic social media use in young adults: A mixed serial-parallel mediation model involving Alexithymia, Defense Mechanisms, and Fear of Missing Out. Cyberpsychology, Behavior, and Social Networking.

[B27-behavsci-16-00802] Groenestein E., Willemsen L., van Koninsbruggen G. M., Kerkhof P. (2024). Fear of missing out and social media use: A three-way longitudinal study on the interplay with psychological need satisfaction and psychological well-being. New Media & Society.

[B28-behavsci-16-00802] Hair J. F., Black W. C., Babin B. J., Anderson R. E. (2010). Multivariate data analysis.

[B29-behavsci-16-00802] Harman H. H. (1976). Modern factor analysis.

[B30-behavsci-16-00802] Holte A. J. (2025). A Structural Equation Modeling Approach to Understand the Dynamics of Smartphone Attachment and Problematic Smartphone Use. Journal of Technology in Behavioral Science.

[B31-behavsci-16-00802] Holte A. J., Aukerman K., Padgett R., Kenna M. (2024a). “Let me check my phone just one more time”: Understanding the relationship of obsessive-compulsive disorder severity and problematic smartphone use. Current Psychology.

[B32-behavsci-16-00802] Holte A. J., Cooper J., Nixon A. (2024b). Burden or refuge? Understanding predictors of smartphone burden and refuge. Current Psychology.

[B33-behavsci-16-00802] Holte A. J., Ferraro F. R. (2020). Anxious, bored, and (maybe) missing out: Evaluation of anxiety attachment, boredom proneness, and fear of missing out (FoMO). Computers in Human Behavior.

[B34-behavsci-16-00802] Holte A. J., Fisher W. N., Ferraro F. R. (2022). Afraid of social exclusion: Fear of missing out predicts cyberball-induced ostracism. Journal of Technology in Behavioral Science.

[B35-behavsci-16-00802] Hou X., Elhai J. D., Hu T., She Z., Xi J. (2023). Anxiety symptoms and problematic smartphone use severity among Chinese college students: The moderating role of social support. Current Psychology.

[B36-behavsci-16-00802] Hu L., Bentler P. M. (1999). Cutoff criteria for fit indexes in covariance structure analysis: Conventional criteria versus new alternatives. Structural Equation Modeling: A Multidisciplinary Journal.

[B37-behavsci-16-00802] Kardefelt-Winther D. (2014). A conceptual and methodological critique of internet addiction research: Towards a model of compensatory internet use. Computers in Human Behavior.

[B38-behavsci-16-00802] Kenny D. A., Kaniskan B. K., McCoach D. B. (2015). The performance of RMSEA in models with small degrees of freedom. Sociological Methods & Research.

[B39-behavsci-16-00802] Kock N. (2015). Common method bias in PLS-SEM: A full collinearity assessment approach. International Journal of e-Collaboration.

[B40-behavsci-16-00802] Koo H. J. (2023). American Idle: An examination of leisure guilt, time use, and well-being. Doctoral dissertation.

[B41-behavsci-16-00802] Kwon M., Kim D.-J., Cho H., Yang S. (2013). The smartphone addiction scale: Development and validation of a short version for adolescents. PLoS ONE.

[B42-behavsci-16-00802] Leary M. R., Kelly K. M., Cottrell C. A., Schreindorfer L. S. (2013). Construct validity of the need to belong scale: Mapping the nomological network. Journal of Personality Assessment.

[B43-behavsci-16-00802] Lee M.-S., Lee H. (2023). Problematic smartphone use and its relationship with anxiety and suicidal ideation among South Korean adolescents. Psychiatry Investigation.

[B44-behavsci-16-00802] Liu C., Yang H., Hao Z., Li J. (2024). The relationship between social anxiety and problematic smartphone use: A chain mediational model. Current Psychology.

[B45-behavsci-16-00802] MacCallum R. C., Hong S. (1997). Power analysis in covariance structure modeling using GFI and AGFI. Multivariate Behavioral Research.

[B46-behavsci-16-00802] Marengo D., Settanni M., Fabris M. A., Longobardi C. (2021). Alone, together: Fear of missing out mediates the link between peer exclusion in WhatsApp classmate groups and psychological adjustment in early-adolescent teens. Journal of Social and Personal Relationships.

[B47-behavsci-16-00802] Marsh H. W., Hau K. T., Wen Z. (2004a). In search of golden rules: Comment on hypothesis-testing approaches to setting cutoff values for fit indexes and dangers in overgeneralizing Hu and Bentler’s (1999) findings. Structural Equation Modeling.

[B48-behavsci-16-00802] Marsh H. W., Wen Z., Hau K.-T. (2004b). Structural equation models of latent interactions: Evaluation of alternative estimation strategies and indicator construction. Psychological Methods.

[B49-behavsci-16-00802] Mehrabian A., Russell J. A. (1974). An approach to environmental psychology.

[B50-behavsci-16-00802] Meng S., Qi K., Huang Y., Shen P., Onyebuchi N., Tong W., Li X., Meng P. (2025). The relationship between anxiety and problematic mobile phone use among Chinese college students: A moderated mediation model. BMC Psychology.

[B51-behavsci-16-00802] Métellus S., Vaillancourt-Morel M.-P., Brassard A., Daspe M.-È. (2025). Attachment anxiety and relationship satisfaction in the digital era: The contribution of social media jealousy and electronic partner surveillance. Journal of Marital and Family Therapy.

[B52-behavsci-16-00802] Modica C. A., Bailey K. (2025). Bidirectional longitudinal associations between emotional regulation, perceived stress, and multiple indices of smartphone use among young-adult women. International Journal of Human-Computer Interaction.

[B53-behavsci-16-00802] Ng S. P., Fam J. Y. (2024). A multidimensional view of fear of missing out as a mediator between the need to belong and problematic smartphone use. Computers in Human Behavior Reports.

[B54-behavsci-16-00802] Nong W., He Z., Ye Y.-H., Wu Y.-F., Wu Y.-T., Ye J.-N., Sun Y. (2023). The relationship between short video flow, addiction, serendipity, and achievement motivation among Chinese vocational school students: The post-epidemic era context. Healthcare.

[B55-behavsci-16-00802] Panova T., Carbonell X. (2018). Is smartphone addiction really an addiction?. Journal of Behavioral Addictions.

[B56-behavsci-16-00802] Park J., Jung Y. (2024). Unveiling the dynamics of binge-scrolling: A comprehensive analysis of short-form video consumption using a stimulus-organism-response model. Telematics and Informatics.

[B57-behavsci-16-00802] Pew Research Center (2025). Mobile fact sheet.

[B58-behavsci-16-00802] Pezzi M., Corsano P., Santoro G., Gori A., Gámez-Guadix M., Musetti A. (2024). Solitary experience and problematic social media use among young adults: A systematic review with recommendations for future research. Clinical Neuropsychiatry: Journal of Treatment Evaluation.

[B59-behavsci-16-00802] Pérez-Torres V. (2024). Problematic use of social media in adolescents or excessive social gratification? The mediating role of nomophobia. Journal of Psychosocial Research on Cyberspace.

[B60-behavsci-16-00802] Piko B. F., Müller V., Kiss H., Mellor D. (2025). Exploring contributors to FoMO (Fear of Missing Out) among university students: The role of social comparison, social media addiction, loneliness and perfectionism. Acta Psychologica.

[B61-behavsci-16-00802] Przybylski A., Murayama K., DeHaan C. R., Gladwell V. (2013). Motivational, emotional, and behavioral correlates of fear of missing out. Computers in Human Behavior.

[B62-behavsci-16-00802] Qu D., Liu B., Jia L., Zhang X., Chen D., Zhang Q., Feng Y., Chen R. (2024). The longitudinal relationships between short video addiction and depressive symptoms: A cross-lagged panel network analysis. Computers in Human Behavior.

[B63-behavsci-16-00802] Savci M., Savci H., Ugur R., Zincirli M., Elhai J. D. (2025). The binge-scrolling scale measures excessive scrolling through a validated three-factor structure. Scientific Reports.

[B64-behavsci-16-00802] Scheier M. F., Carver C. S., Bridges M. W. (1994). Distinguishing optimism from neuroticism (and trait anxiety, self-mastery, and self-esteem): A re-evaluation of the Life Orientation Test. Journal of Personality and Social Psychology.

[B65-behavsci-16-00802] Shannon H., Mongomery M., Guimond S., Hellemans K. (2025). Problematic social media use and inhibitory control among post-secondary students. Addictive Behaviors.

[B66-behavsci-16-00802] Świątek A. H., Szcześniak M., Bielecka G. (2021). Trait anxiety and social media fatigue: Fear of missing out as a mediator. Psychology Research and Behavior Management.

[B67-behavsci-16-00802] van den Eijnden R. J. J. M., Lemmens J. S., Valkenburg P. M. (2016). The Social Media Disorder Scale. Computers in Human Behavior.

[B68-behavsci-16-00802] Wang L. (2025). Are you watching people via social media?. Master’s Thesis.

[B69-behavsci-16-00802] Wickord L.-C., Quaiser-Pohl C. M. (2022). Does the type of smartphone usage behavior influence problematic smartphone use and the related stress perception?. Behavioral Science.

[B70-behavsci-16-00802] Wong B. (2023). Top social media statistics and trends of 2023.

[B71-behavsci-16-00802] Wu Y., Zhou W., Liu F., Meng X., Wang F. (2026). Daily dynamics of rest intolerance, anxiety, and problematic smartphone use: Insights from 14-day intensive longitudinal study. Personality and Individual Differences.

[B72-behavsci-16-00802] Xu C., Gu Z., Yan J., Lock M., Chen S., Zhuang Q. (2025). The separation of adult ADHD inattention and hyperactivity-impulsivity symptoms and their association with problematic short-video use: A structural equation modeling analysis. Psychology Research and Behavior Management.

[B73-behavsci-16-00802] Yao N., Hallauer C. J., Hutcheson E. F., Elhai J. D. (2025). Fear of missing out mediates the relationship between intolerance of uncertainty and problematic smartphone use severity. Cyberpsychology: Journal of Psychosocial Research on Cyberspace.

[B74-behavsci-16-00802] Zeba F., Hollebeek L. D., Kesharwani A., Shaheen M., Arvola R. (2026). FoMO-driven binge-scrolling and eudaimonic wellbeing. Journal of Retailing and Consumer Services.

[B75-behavsci-16-00802] Zhan Y., Luo L., Hu X. (2025). Digital stress and problematic social media use among college students: Exploring dual emotional pathways and the moderating role of cognitive reappraisal. BMC Psychology.

[B76-behavsci-16-00802] Zhang N., Hazarika B., Chen K., Shi Y. (2023). A cross-national study on the excessive use of short-video applications among college students. Computers in Human Behavior.

[B77-behavsci-16-00802] Zhao Z., Kou Y. (2024). Effect of short video addiction on the sleep quality of college students: Chain intermediary effects of physical activity and procrastination behavior. Frontiers in Psychology.

